# Understanding collaborative care implementation in the Department of Veterans Affairs: core functions and implementation challenges

**DOI:** 10.1186/s12913-017-2601-9

**Published:** 2017-10-10

**Authors:** Jessica M. Lipschitz, Justin K. Benzer, Christopher Miller, Siena R. Easley, Jenniffer Leyson, Edward P. Post, James F. Burgess

**Affiliations:** 1 Center for Healthcare Organization and Implementation Research, VA Boston Healthcare System, 150 South Huntington Ave, Jamaica Plain,, Boston, MA 02130 USA; 2 Harvard Medical School, Department of Psychiatry, Boston, USA; 3 Department of Health Policy and Management, Texas A&M University School of Public Health, College Station, USA; 4 Central TX VA Healthcare System, VISN 17 Center for Research on Returning Veterans, Temple TX, USA; 5 VA Office of Primary Care Services, Ann Arbor, USA; 6 Boston University School of Public Health, Department of Health, Law, Policy and Management, Boston, USA; 7 University of Michigan Medical School, Department of Internal Medicine, Ann Arbor, USA; 8 VA Ann Arbor Healthcare System, Center for Clinical Management Research, Ann Arbor, USA

## Abstract

**Background:**

The collaborative care model is an evidence-based practice for treatment of depression in which designated care managers provide clinical services, often by telephone. However, the collaborative care model is infrequently adopted in the Department of Veterans Affairs (VA). Almost all VA medical centers have adopted a co-located or embedded approach to integrating mental health care for primary care patients. Some VA medical centers have also adopted a telephone-based collaborative care model where depression care managers support patient education, patient activation, and monitoring of adherence and progress over time.

This study evaluated two research questions: (1) What does a dedicated care manager offer in addition to an embedded-only model? (2) What are the barriers to implementing a dedicated depression care manager?

**Methods:**

This study involved 15 qualitative, multi-disciplinary, key informant interviews at two VA medical centers where reimbursement options were the same— both with embedded mental health staff, but one with a depression care manager. Participant interviews were recorded and transcribed. Thematic analysis was used to identify descriptive and analytical themes.

**Results:**

Findings suggested that some of the core functions of depression care management are provided as part of embedded-only mental health care. However, formal structural attention to care management may improve the reliability of care management functions, in particular monitoring of progress over time. Barriers to optimal implementation were identified at both sites. Themes from the care management site included finding assertive care managers to hire, cross-discipline integration and collaboration, and primary care provider burden. Themes from interviews at the embedded site included difficulty getting care management on leaders’ agendas amidst competing priorities and logistics (staffing and space).

**Conclusions:**

Providers and administrators see depression care management as a valuable healthcare service that improves patient care. Barriers to implementation may be addressed by team-building interventions to improve cross-discipline integration and communication. Findings from this study are limited in scope to the VA healthcare system. Future investigation of whether alternative barriers exist in implementation of depression care management programs in non-VA hospital systems, where reimbursement rates may be a more prominent concern, would be valuable.

## Background

Mood disorders are widespread, with a lifetime prevalence of 20.8% in the U.S [1]. Effective treatments with moderate effect sizes do exist [2]. However, the proportion of individuals who seek treatment for this condition ranges widely from 13 to 57%, leaving about half of the population untreated [3, 4]. The cost of untreated depression in the United States is estimated at tens of billions of dollars annually [5]. Reasons frequently cited for not seeking care include not knowing one has a treatable illness [6], not acknowledging symptoms are severe enough to warrant treatment [7], having negative attitudes toward treatment [8], getting lost in the referral process [9] or the mental health specialty system, and experiencing stigma related to mental health services [10].

One strategy for increasing depression treatment rates is to provide access to integrated mental health care. Integrated mental health care refers to a set of models that consider patients’ medical and psychological treatment needs. One prominent, evidence-based method of integrating mental health care is the Collaborative Care Model [11]. The Collaborative Care Model uses care management to regularly contact patients and collaborate with medical doctors and mental health specialists to organize patients’ care [12]. The key innovation of the Collaborative Care Model is the use of a dedicated care manager, commonly a nurse or social worker, who coordinates psychological treatment and monitors progress [13]. The Collaborative Care Model has strong effects in terms of both patient satisfaction [14] and depression outcomes [15–19].

### Collaborative approaches to depression treatment in VA

The Department of Veterans Affairs (VA), along with other healthcare systems, has adopted a general ‘collaborative approach’ to healthcare. A central part of this collaborative approach is the VA medical home model called ‘Patient-Aligned Care Teams’ that is designed to be consistent with the Chronic Care Model [20]. While VA has mandated that all VA medical centers provide ‘integrated mental health care’ as part of these Patient-Aligned Care Teams, facilities are given substantial discretion about structural features of that integration. The most common model is to embed mental health practitioners in the primary care setting to increase access to mental health consultation, education, assessment and brief treatment such as short courses of psychotherapy [21, 22]. These co-located or embedded models of mental health integration generally espouse a collaborative approach to mental health care. This approach usually fosters nominal collaboration through collegial relationships [23] Conversely, some sites have chosen to implement a model that adds dedicated telephone-based depression care managers to the team of embedded mental health practitioners. These mental health practitioners supervise the care managers as well as independently provide direct services co-located within the primary care setting, and this is referred to as a blended models by VA administrators (i.e., blending embedded mental health care with depression care management). For purposes of this paper, we will refer to this scenario that adds systems approaches as the care management (CM) model. The CM model is consistent with the Collaborative Care Model. We do note that outside of VA, the use of embedded mental health staff in addition to care managers is not necessarily a component of CM. In VA, the evolution of integrated care almost always began with embedded providers and thus CM sites typically include both dedicated care managers (often off-site) along with other embedded mental health staff.

It is unclear why some VA medical centers choose to implement only the embedded model, and do not choose to implement the dedicated care manager role that is the hallmark of the Collaborative Care Model. One possible explanation is a perception that treatment quality has been maximized and thus hiring a dedicated care manager is unnecessary. The Collaborative Care Model for depression most often incorporates a set of the following functions: motivational interviewing and behavioral activation [24–26], relapse prevention [14]; monitoring of symptoms and medication adherence [9, 24–32]; supportive discussions of self-management [14], and goal-setting [24, 26]. However, these care management functions could be performed by co-located/embedded mental health professionals and perhaps a dedicated care manager is unnecessary at these sites. A second hypothesis is that there are cultural, financial, or organizational barriers to implementing a dedicated care manager. Despite the perceived benefits of a dedicated care manager, these barriers may limit adoption. The current study compared a site that implemented dedicated care managers to a site that had implemented the embedded model (without dedicated care managers). Specifically, this qualitative study focused on two main research questions.

### Research questions (RQ)

#### RQ1: What does a dedicated care manager offer in addition to an embedded model?

In theory co-located models could provide many or all of the same core care management functions as a dedicated care manager. Therefore, we sought to identify what administrative leaders and primary care and mental health professionals perceived as the core care management functions of an integrated mental health model, and compare the functions provided by a dedicated care manager to the functions provided in an embedded model.

#### RQ2: What are the barriers to implementing a dedicated care manager?

Despite both documentation of the burden of depression on the primary care system and research supporting improved outcomes with integrated mental health care models that include care management, such programs are not yet widespread [22, 33, 34]. Research on reasons for slow implementation is currently limited. Existing research suggests provider engagement [35, 36]; communication by telephone with difficulties across scheduling, commitment, convenience [14]; and reimbursement methods [31] are barriers to effective program implementation. Therefore, we sought to identify the barriers experienced implementing a dedicated care manager, as well as the implementation barriers perceived at a medical center with an embedded model.

## Methods

This study used thematic analysis of qualitative multi-disciplinary key informant interviews at two VA medical centers. This research was approved by the Boston VA Institutional Review Board (IRB approval #2754). Informed consent was discussed at the beginning of all interviews and included a discussion of publishing qualitative interview data.

### Research team

All interviews were conducted by at least two PhD-level authors. In all cases, at least one interviewer was experienced in qualitative research. All authors were VA employees and full-time health services researchers. Most authors had either clinical or research experience with the VA PC-MHI program. No authors had previous relationships with the study sites.

### Study site and sample selection

Staff participants were recruited by email from two VA medical centers, one with blended collaborative care (in this case co-located mental health practitioners and dedicated care managers) and one with co-located mental health practitioners, but no care managers. Both sites receive capitated payments in which mental health diagnoses provide additional funding.

Potential sites were identified though survey data of VA Primary Care-Mental Health Integration (PC-MHI) programs and consultation with national program managers to identify sites with strong implementation of program characteristics [37, 38]. We selected one site that had implemented a dedicated care manager (hereafter CM) because it was a national leader in both depression care management and in implementation of embedded mental health care. For comparison, we selected one site that had implemented only embedded mental health staff (hereafter EMBED) because it had a well-organized PC-MHI program but had not yet implemented a dedicated care manager. Potential participants were identified by national program leaders, internal VA lists (i.e., websites and mail groups), and snowball sampling to adjust for unreliability in VA lists. Recruitment continued until consensus was reached that data saturation was achieved. Recruitment emails were sent to 45 individuals, 15 of whom agreed to participate. Nonresponse was higher at the EMBED site. Employees were not asked about reasons for nonparticipation, but leader interviews at the EMBED site indicated a number of competing administrative priorities that may have contributed to lower response rate. Interviews were conducted first with leaders to gain both access and an understanding of their program structure. Next, we interviewed care management and mental health staff to identify core program functions. Finally, we interviewed primary care staff to understand their role in depression treatment (See Table 1 for summary of participants).

### Data collection

Thirty-minute interviews were conducted by telephone with the participants at a private location in their workplace, digitally recorded, transcribed by study staff, and input into NVIVO 8 for analysis. No repeated interviews were conducted, and neither transcripts nor findings were presented to participants. Interview questions were pilot tested with PC-MHI staff at the study team’s home VA site.

Semi-structured interview guides began with a “grand tour” question about the process of caring for a patient with depression, and then probed regarding the care management functions. This design allowed participants to talk about depression care using their own language and could have revealed other core care management functions not expected. Interview guides were modified for each site and for different types of informants. For example, only the primary care participants at the CM site were asked, “What happens to patients after you refer them to care management?” When appropriate, participants were asked to evaluate current practices by comparing current performance to ideal performance, identify facilitators of success, and identify barriers. All participants were asked specific questions about (1) how their program encourages patients to be actively involved in their mental health care, (2) how patients are educated regarding mental health treatment, and (3) processes for monitoring patients with depression over time.

### Coding

One experienced qualitative researcher unfamiliar with the conceptual framework first performed open coding of the CM site interviews. This allowed the team to identify any potential care management core functions provided in the CM site that were not expected. The study team reviewed the open codes and confirmed that the three care management functions were sufficient. The research team reached consensus on uniqueness and clarity for the open codes, generating 25 program structure codes, 21 care management function codes, 35 program implementation codes, 3 program evaluation codes, 3 program outcomes codes, 5 benefits to patients codes, 10 codes about how care management affects primary care providers, 13 codes about coordinating depression care in general, and 7 additional codes (e.g., details about the primary care program structure). Open codes were consistent with the conceptual framework. No additional concepts were identified for analysis.

Two investigators read and coded/re-coded transcripts from both sites using the following codes: patient education, patient activation, monitoring of adherence and progress over time, inter-professional communication, barriers to care management, facilitators of care management, and program descriptions. A third investigator reviewed all codes. Disagreements were resolved through discussion. The constant comparative method was used to revise conflicting codes and develop clear conceptual definitions grounded in both the data and the literature [39].

### Analysis

Thematic analysis was used to identify patterns in both the descriptive and the analytical themes related to how care management was discussed [40]. *Descriptive themes* are summaries of the content of coded data. For example, one descriptive theme for the code “patient activation” was “care managers encouraging patients to pursue self-care goals.” *Analytical themes* were developed by comparing descriptive themes from the CM site to those from the EMBED site. For example, descriptive themes regarding staffing were reported in each site, but the CM site discussed how to hire the best care managers, whereas the EMBED site discussed barriers to hiring. Themes were developed through an iterative process of comparing coded data within and between sites, reading the data in the context of the original interviews, memoing [41], and discussing the themes as a team until consensus was reached. To answer RQ1, codes for the care management functions were compared between sites. To answer RQ2, each site was analyzed separately. The CM site identified barriers that arose during the implementation process. The EMBED site explained why a care management program had not yet been implemented.

## Results

### Characteristics of collaborative care

Table 2 summarizes the descriptive themes regarding the program characteristics at each site. Co-located mental health practitioners at each site (i.e., psychologists, psychiatrists, nurses, and/or care managers) support treatment for patients in primary care by providing assessments, developing treatment plans, managing referrals, prescribing psychiatric medications, and being available for same-day consultation. Elements of collaboration such as sharing common tasks and joint clinical decision-making may vary.

**Table 1:** Recruited Participants’ Characteristics

	CM Site (1) *n* = 9	EMBED Site (2) *n* = 6
Leadership	2	1
Primary Care Clinicians^a^	3	2
Care Management Staff	2	2^b^
Mental Health Staff	2	1
Gender	Male: 3; Female: 6	Male: 1; Female: 5

*Notes*: ^a^Includes nurses and physician assistants, ^b^These care management staff were from the site’s telehealth program

**Table 2:** Descriptions of Integrated Primary Care System

Theme	CM Site	EMBED Site
Same Day Access	“And then we also have NPs, psychiatric NPs available, 2 or 3, depending on our staffing levels, who see walk-in patients with medication needs, and they also refer to care management” [Primary Care Psychologist] “In the VA system I’m in, they have a behavioral health counselor…if I feel they need to be seen in Psychiatry, I filter through her. And it’s actually fabulous. She is right in the same building as me. I can say, “Hey, BHC. Can you do me a favor? This guy, I’m afraid if we send him home right now, he’s going not follow up,” and she says, ‘I’ll get that at two o’clock. I’ve got a one o’clock. Tell him whatever.’ Fabulous - she works him in.” [Primary Care Provider]	“We actually have a mental health nurse practitioner… within our primary care clinic and they are available to see patients who really need, you know, mental health evaluation or intervention that day.” [Physician’s Assistant] “…a primary care patient…gets screened by the LPN when they come in…they asked the generic questions of depression screening…if they trigger positive then they let us know as the providers….if they seem like they need to be referred or if they seem like they are at risk immediately, then we have the luxury…of having a nurse practitioner and a mental health team available in our primary care area. So we can…evaluate that patient the same day.” [Nurse Practitioner]
Support for Primary Care Providers around mental health assessments, referrals, treatment planning, and psychiatric medications	“…[referring to an embedded psychologist] then she assesses, ‘Hey, [PCP], I think maybe you were overestimating,’ or ‘Yeah, I think you’re accurate. This person needs Psychiatry, this person needs SATP [Substance Abuse Treatment Program], this person should be treated at the PTSD clinic.” [Primary Care Doctor] “So I’m an embedded or co-located MH provider in PC, and so each one of our PC [providers] …has one behavioral health provider embedded, and so we all would refer to the care management program.” [Primary Care Psychologist]	“…so if the primary care provider can’t or doesn’t feel comfortable managing the depression…then they would contact the primary care mental health nurse practitioner.” [Physician’s Assistant] “So, if the provider, the primary care provider, is, you know, after they assess the patient and if they’ve determined that this is something that can be managed, that they’re comfortable managing in primary care, that’s one route that they’ll go. And so, you know, they would prescribe the med and then set up a follow-up, typically phone with their PACT nurse” [Physician’s Assistant]

*Note. BHC* behavioral health counselor, *PCP* primary care provider, *MH* mental health, *PACT* patient aligned care team

The CM site has a care management program in which patients can be referred based on their scores on the PHQ-9 [42] or the GAD-7 [43], well-validated depression and anxiety screening tools, respectively. Care management here involves monthly calls focused on educating patients about depression and related behavioral health concerns, keeping patients engaged in their treatment, monitoring progress (or deterioration) of the condition in the context of overall health, and facilitating changes in treatment (e.g., antidepressant dosing). The clinical focus of these calls is typically depression, anxiety, medication adherence and sometimes other health behavior change goals such as smoking cessation. The number of calls attempted is determined based on PHQ-9 and GAD-7 scores.

### What does incorporating a dedicated care manager offer?

The Chief of Mental Health summarized why care management is valuable for engaging and tracking patients between scheduled in-person visits:

*“I think [the Veterans] just appreciated the fact that there were people checking up on them. There's kind of a trust that you build, and there's a comfort from them knowing that you care and you're actually trying to help them that goes a long way towards helping them be as compliant as they can be. And again, there's also the points of information because many months can go by between our visits, to just have another data point from where they are is always valuable.” [CM site]*

At the CM site, informants described how the program provides core functions of patient activation, patient education, and monitoring of patients over time.

#### Patient activation

Participants identified emotional support as a central part of patient activation in the care management program. Participants felt that evening and weekend contact keeps patients engaged in treatment and communicates caring. Care managers believed that calls motivated patients to pursue individual health goals between in-person meetings. One nurse Care Manager provided a typical example of developing an emotionally supportive relationship with a patient:

*“The first time [care manager] called him this morning he said, ‘Why in the hell are you calling me? I’ve got my hands full…,’ and just extreme anger. Well, he’s overwhelmed with everything. And she just said, “Look you don’t need another aggravating phone call at this point,” and just kind of calmed him down and just said, “…I know you don’t want to talk to me and that, but what I can do is I can just call back in a couple of weeks and just see how you’re doing,” and he acquiesced to that”[CM site]*

Another nurse described how the program fosters independent motivation for patients to reach their own health goals:

*“Well, during that initial call, they are asked for a kind of what their goal is, what they want assistance with in improving in their life... So, I don't want to say it's a treatment plan. It's kind of a quasi-treatment plan where the veteran develops what their goal is, and then the Care Manager will help facilitate that, you know, in whichever way. We also really emphasize a recovery-based philosophy, and trying to instill a sense of independence with these Veterans and not dependence that we're going to be calling you forever.” [CM site]*

#### Patient education

Participants described patient education as a central part of care management call content. Areas of education identified include information on medications, potentially useful services, and how to self-manage behavioral health concerns (e.g., improving sleep, exercise goals, stress management, pain management, smoking cessation, and relaxation techniques). As part of the program, psychoeducational packets are sent out to Veterans based on depression and anxiety scores.

The importance of patient education in the care management program was discussed by several informants.

*“On all their calls, [care managers] do health education, whether it's symptomatology or about their medications or coping strategies or healthy living strategies. Those are all discussed with the veterans.” [CM Site]*

#### Monitoring of adherence and progress over time

Participants discussed how care managers use validated measures to monitor depression (i.e., PHQ-9) and anxiety (i.e., GAD-7) symptoms. A care management nurse described how scores on these measures affect both the length of the monitoring process and whether care is managed by licensed practical nurses (LPN) or registered nurses (RN).

*“If their score on the PHQ-9 is 9 or below and the GAD-7 is 9 or below, generally they will stay with the LPN and go through what we call ‘the watchful waiting.’ And they will stay in the program for approximately 3 months…she’ll conduct the PHQ-9 and the GAD-7 with them, and … a few more questions about, you know, if they’re on alcohol …if their scores are 9 or above, then they’re turned over to one of the RNs, and then we would follow them for a period of 6 months. And we would discuss medications, side effects if they’re having any, any resources available, go through coping skills, that type of thing.”[CM site]*

### Are care management functions being carried out at the EMBED site?

Interviews indicated that many of the functions the study team identified as ‘care management’ are provided at the EMBED site. However, the care management functions are less organized compared to the CM site and they depend entirely on individual primary care providers’ knowledge of existing programs and their inclination to refer patients to these programs. Supporting this theme, individual providers reported working to activate patients within the constraints of limited resources. One Primary Care Nurse Practitioner described doing “our best” to activate patients:

*“And so sometimes, depending on the patient, if we know them well, we can use motivational interviewing to help them move on the continuum, regardless of the diagnosis. Whether it be eating less salt or whether it be, you know, leaving the house to get groceries. If we can figure out how to help them do that in a comfortable safe manner, then we will do that in primary care. We won’t just say, ‘Oh they need a mental health referral.’ Because, our resources are pretty tight right now for mental health. So we’ll do our best in primary care to fix that, you know, to help them.” [EMBED site]*

However, access to specific care management functions is limited at the EMBED site. With regard to patient education, a Mental Health Nurse was asked, “if you were able to design an ideal process for getting patients involved, is there anything you would change about your current system?” She responded:

*“Well, certainly quicker access with [an] educational session right away.”[EMBED site]*

Participants expressed that monitoring is not always possible and very much depends on the preferences and capabilities of the particular provider. For example, when asked about monitoring symptoms of depression, one Physician’s Assistant stated:

*“We are relying on the mental health nurse practitioner to do things that really…we should have a care manager in there. Really, I mean what’s the ideal? I would say we have an RN or a social worker also embedded within our primary care clinics to follow the people who maybe they’re newly being started on their antidepressant or who for whatever the reason the primary care team feels, just needs a little extra following a while.” [EMBED site]*

In general, therefore, participants indicated that the current system could benefit from more resources, including a formal care manager supporting treatment for patients more consistently across providers.

### What are the barriers to implementation of a dedicated care manager?

#### Impediments to optimal functioning

Participants at the CM site articulated barriers to running an effective care management program as (1) staffing; (2) integration and collaboration; and (3) primary care provider burden.

##### Staffing

Participants commented on the challenge of finding care managers with the specific interpersonal qualities required. Interviewees at the CM site noted that care managers must be warm to encourage patient participation and aggressive in communicating and collaborating with primary care providers and PACT staff.

##### Integration and collaboration

The most frequently mentioned barriers to care management involved creating environments for integrating information across services and collaboration between primary care and mental health.

One issue related to collaboration was the visibility of care managers. Participants expressed that the program would be more effective if they were more involved in routine activities like staff meetings. Participants indicated that without such integration care managers are marginalized in primary care teams, which can limit their effectiveness. In this vein, the Mental Health Chief suggested how care managers could be best implemented elsewhere.

*“I think what I would want to do is really pair the care managers with the rest of Primary Care/Mental Health integration staff. What happens is since this is telephone monitoring, it’s very easy not to include them in the regular meetings because they are contacting patients by phone, they’re not there, visible on a face-to-face basis with patients, and so now I know that engaging them and placing them as part of the team, an active participant with input in that they need to be in meetings, face-to-face meetings with the rest of the providers, talking about some of the challenges and dilemmas.”[CM site]*

A similar issue was care management program visibility. Participants reported that primary care providers need to have a clear understanding of when and how to engage the program within their existing complex workflows. Interviewees thought that this can be difficult for primary care providers. An embedded psychologist at the CM site explained,

*“PCPs in general are very confused about all of these mental health programs, and they don’t have any idea what’s what…they don’t know the difference between depression care manager, me the embedded provider, and a primary care evaluation by the psychiatric nurse practitioner. And we’re constantly trying to educate them about it, but we have turnover of providers and they’re very, very busy.”[CM site]*

This suggests an important system-level deficiency in support for integrated mental health. Education of individual primary care physicians is challenging in general, and the effectiveness is further limited by high workload and turnover.

Another key theme related to collaboration was that of the relationship between primary care providers and care managers, both in terms of conflict and trust. Conflicts focused on division of labor and work tasks. According to one nurse care manager,

*“It’s just you know, the old, you know, the war between primary care and mental health and that, you know, they just don’t want to work together.”[CM site]*

Primary care providers also articulated difficulties letting go of responsibility for all aspects of patient care and trusting that important information would be brought to their attention. One primary care provider stated,

*“I have difficulty saying, ‘I’m going to start you on this medication and I’m going to have somebody follow this.’ I’m having difficulties with letting it go. …my natural inclination is to say, ‘OK, I’m going to start you on this medicine. I’m going to see you in four months and follow up’…part of my reluctance… I just don’t have that trust, I guess, of that system yet.”[CM site]*

Overall, these themes suggest the need for improved systems and team functioning, even in these sites that were identified as high performing examples. Visibility of care managers and the care management program, as well as provider-level conflict and trust seemed to be barriers to optimal functioning.

##### Primary care provider burden and knowledge

Many participants expressed the view that the system places extra burden on primary care providers. First, the growing volume of information exacerbates administrative overload. One primary care provider stated,

*“The question is how much time do you have to deal with those multiple opportunities on many patients when there's also the rest of the business of the day to attend to…there were times I would think, "Boy, here's this point of information. I'm already trying to deal with all these other points of information about other patients…it could easily overwhelm the capacity to deal with everything that we deal with on a day-to-day basis.”[CM site]*

Further, one primary care provider expressed that information from the care management program is not flagged in a way that lets him know what is most important,

*“I get anywhere from 40-50 [computer alerts] a day, so you can imagine how many I have on my dashboard. So I get a telephone note from a nurse about something that's sandwiched in among the other 40 or 50 [computer alerts]. Well, it's sandwiched in there with the abnormal stress test and the lung mass that was seen, and it's tough to be able -- I would like a way where I could get communication saying, ‘Hey listen, this person, it isn't working. They have this side effect. They have a problem,’… getting the information to whatever provider needs to deal with it sometimes is a problem.” [CM site]*

#### Impediments to moving beyond an embedded model

Many participants in the EMBED site expressed that care management would strengthen services provided. For example, a Physician’s Assistant stated,

*“You know, specialty mental health needs to be there for the patients who need that more intensive mental health evaluation and treatment and if we’re clogging up mental health access with simple, you know, kind of uncomplicated depression then we’re not going to be able to get the patient in who really needs that level of care…You have to build in the specialty of that mental health support for the primary care providers if we’re going to be successfully getting them to treat the uncomplicated depression.”[EMBED site]*

Nevertheless, participants articulated two key barriers to getting such a program started: (1) getting care management on leaders’ agendas; and (2) logistics—specifically, staffing and space.

##### Getting care management on leaders’ agendas

On a general level, interviewees at the EMBED site expressed that care management programs can be implemented only when site leaders are given time to look at the bigger picture and respond to demands on the system. Interviewees commented that with public scrutiny on the VA regarding immediate operational issues, it can be difficult for leaders to focus on longer-term plans for improving care delivery. For example, an administrative leader referred to the disruption around making transformative changes caused by frequent inquiries by the media and government representatives:

“*Let me give you this example: Do I respond to a [request for information] or a, you know, whatever crisis there is? Or you know, do some of this longer-term work?”[EMBED Site]*

Similarly, a primary care clinician stated that leaders’ attention has been focused on pressing public relations issues, which has made attention to new programming difficult:

*“We’ve really been focusing a lot of our time on the issues that have been out there in the media. So, we’re still moving forward, you know, with primary care mental health integration, but that’s really been probably the biggest local factor that has just stalled us a little bit for the last couple months.” [EMBED Site]*

##### Logistics: Staffing and space

Staffing and space were mentioned as key barriers to establishing a formal care management program. Prior research on PC-MHI implementation on VA had revealed similar issues with regard to the difficulties in hiring personnel and finding the space needed for those staff [21, 44]. According to a mental health leader,

*“…for example, we have one nurse practitioner who does triage for the Urgent Care, and consults for the Medical Unit, and Primary Care Mental Health Integration for numerous PACTs. And so there’s no way you’re gonna get that person to do real care management on top of that.” [EMBED site]*

Similarly, a Physician’s Assistant stated that current space was not sufficient to hire and deploy staff that are integrated:

*“…space has been one of our biggest challenges...the people that we’re actually hiring are not, at this point, even going to be able to be in the clinic because we don’t have space for them. So we’re going to have to get creative in how to make sure they feel tied to those clinics even though they’re not physically located there.” [EMBED site]*

Overall many barriers to establishing care management appeared to be administrative, rather than provider-driven.

## Discussion

This research identifies challenges to integrating depression care into primary care both in sites with dedicated depression care managers and in sites without formal depression care managers. Two functions of care management, patient education and activation, can be provided through embedded programs. The clearest gap is in monitoring of progress over time, and the attention to systems issues that will assure that monitoring. However, implementing a care management program may introduce additional challenges to integration. This research suggests that specific attention to integration in regards to program structure (e.g., dissemination of program knowledge), interpersonal relationships (e.g., trust), and communication systems (e.g., computer alerts) is essential to the reliability of care management functions. Furthermore, findings indicate that administrative factors (staffing and space), provider burden and competing priorities are key challenges for implementing care management. Results therefore indicate that administrators should be clear about the need for enhanced monitoring over time before implementing a depression care management program. If primary care patients only require more patient education and activation, it may be as effective to enhance resources for these functions with embedded mental health staff.

The need for accessible depression treatment increasingly is acknowledged as a broad healthcare problem. Primary care-based care management programs for depression offer a scalable solution to part of this problem and are being implemented in a variety of healthcare systems, including many VA medical centers. Key informants from a site offering both co-located care and care management saw many benefits of this blended model of integration. Providers and administrators reported that the care management program creates a structure for routine monitoring of depression and anxiety and provision of key components of behavioral health interventions (e.g., psychoeducation). They also expressed that the frequent and personalized contact provided by the program helps motivate patients to get and stay engaged in their depression treatment plan.

In contrast, participants from a site with a collaborative approach to co-located care, but without care management, specified a number of significant gaps in services offered. They stated that monitoring patients with depression between scheduled, in-person appointments is not conducted in a systematic way at their site. Specific care management functions were available, but without consistency or cohesion. They specified that quicker (i.e., same day) and enhanced (i.e., more intervention components like patient education) treatment would benefit patients. Finally, they suggested that having a care management program would allow more attention to and follow-up on patient concerns.

The findings regarding barriers to care management program implementation also lend insight into potential intervention targets for program implementation. Feedback on care management implementation barriers was unified around the theme of the difficulty operating successfully as an efficient, multi-disciplinary team. Specifically, interviewees at the CM site expressed difficulty in finding ways to work collaboratively, which led to increasing rather than decreasing providers’ workload. They attributed collaboration difficulties to insufficient computer systems for flow of critical information between care managers and providers, tension in the inter-professional relationship (e.g., providers not trusting care managers to follow up on prescriptions and being dismissive of care managers), and poor on-site visibility of care managers. Interestingly, the theme of team collaboration was also raised at the EMBED site in terms of a need for “intact or functioning PC-MHI teams” and “support for the primary care providers.”

One interpretation of these findings is that interventions focused on interdisciplinary team cohesion, communication, and specific task behaviors may be successful at improving functioning of collaborative care programs whether or not they involve care management. More specifically, such an intervention could include logistical components that help to improve interaction, such as provision of on-site office space for all team members and holding staff meetings with all team members across disciplines. It should provide educational components to cultivate a strong understanding of available programs, and promote joint attention to care pathways and specific expectations for providers’ respective roles. Finally, it could include a technological component: building clearer electronic alert systems for urgent patient information. Sites could regularly measure patients’ perceptions of education, activation, and monitoring in order to determine the need for either stronger support from embedded mental health staff or justify the need for investment in a depression care management program.

Interviewees in the EMBED site indicated that leadership priorities and logistics are barriers to implementing a care management program. Other research has shown that these are common barriers in implementing care management [45]. These barriers seem potentially surmountable if resources and administrative focus were shifted. Potentially, awareness of the strong evidence that care management programs improve patient satisfaction and health outcomes and reduce costs could facilitate such shifts. In that regard, future research might productively study how to influence the adoption and implementation of collaborative care models.

This study has several limitations. First, it was limited in scope: interviews were conducted exclusively within the VA healthcare system and only at two sites. However, similar barriers have been found in other healthcare systems [45]. These sites represent specific points on a formative journey toward collaborative care models but do not represent all VA facilities’ approaches to integrated care. Further research in VA as well as studies at non-VA systems are warranted. Second, interviews were voluntary, so all participants recruited were interested in being part of a research study and discussing collaborative care. Most interview offers were accepted at the CM site. At the EMBED site, we reached data saturation. However, this required reaching out to many potential participants who refused to participate. Therefore, selection bias may have occurred, especially at the EMBED site. Third, patients were not interviewed. It is possible that patients may experience depression care management in ways that are not intended or articulated by healthcare staff. Fourth, no quantitative data were used to contextualize the sites. We used judgments of administrators and researchers to select sites rather than quantitative measures. Finally, both sites had a co-located care program. Additional research in settings without embedded mental health providers would lend further insight into implementation barriers.

Results from this study support a number of directions for future research. First, the current study was qualitative and did not measure patient outcomes or interview patients for their perspective on the program. Future studies could conduct a similar comparison (i.e., CM site versus EMBED site) to test differences in variables such as patient satisfaction, remission from depression, and budget impact that would strengthen the case for the value of care management. Additionally, as mentioned above, this study was conducted exclusively within the VA system, which does not face the same reimbursement system challenges that other care providers face. While the VA has been a leader in research and implementation of care management programs, operationalizing potential differences in care management programs within and outside the VA would be useful as healthcare systems seek to design and implement optimal care management programs for their unique settings. Finally, building upon findings that lack of interdisciplinary collaboration is a barrier to effective implementation, future research could evaluate whether an intervention focused on team-building and specific task behaviors improves collaborative care both with and without care management.

## Conclusion

Findings suggest that the care management component of collaborative care models may provide a reliable system to ensure patient education, activation and monitoring of primary care-based depression treatment. However, this study indicates that depression care management creates additional challenges to teamwork beyond the challenges introduced by embedded mental health staff. Difficulties exist in implementing care management programs both because they require resources (e.g., leaders’ attention, space, appropriate staff) and a systems-based approach in terms of collaboration, communication of information, and clinical oversight embedded into care processes and inherent in an interprofessional approach to care. Findings support implementation of primary care-based care management for depression and use of team-building strategies for increasing the effectiveness of implementation. Future research evaluating interventions aimed at more effective implementation of teamwork in depression care management programs and the patient experience of integrated mental health care is needed.

## Abbreviations

BHC: behavioral health counselor
CM: collaborative care model/care manager
EMBED: embedded/co-located care
GAD-7: generalized anxiety disorder 7-item scale
LPN: licensed practical nurse
MH: mental health
PACT: patient aligned care team
PC-MHI: primary care/mental health integration
PCP: primary care provider
PHQ-9: patient health Questionaire-9
RN: registered nurse
RQ: research question
VA: Veterans Affairs
